# A Peptide-Based Assay Discriminates Individual Antibody Response to the COVID-19 Pfizer/BioNTech mRNA Vaccine

**DOI:** 10.3390/vaccines9090987

**Published:** 2021-09-03

**Authors:** Immacolata Polvere, Serena Voccola, Alfredina Parrella, Gaetano Cardinale, Lucrezia Zerillo, Romualdo Varricchio, Jessica Raffaella Madera, Romania Stilo, Pasquale Vito, Tiziana Zotti

**Affiliations:** 1 Dipartimento di Scienze e Tecnologie, Università Degli Studi del Sannio, Via dei Mulini, 82100 Benevento, Italy; immapolvere88@gmail.com (I.P.); lzerillo@unisannio.it (L.Z.); jrmadera@unisannio.it (J.R.M.); romsti@unisannio.it (R.S.); 2 Genus Biotech, Università Degli Studi del Sannio, SS Appia, 82030 Apollosa, Italy; serena.voccola@tecnobios.com (S.V.); romualdovar@outlook.it (R.V.); 3 Consorzio Sannio Tech, SS Appia, 82030 Apollosa, Italy; alfredina.parrella@tecnobios.com (A.P.); gaetano.cardinale@tecnobios.com (G.C.)

**Keywords:** COVID-19, vaccine, antibodies, ELISA, peptides

## Abstract

The coronavirus disease 2019 (COVID-19) mRNA vaccine developed by Pfizer/BioNTech has been shown to be capable of developing an excellent antibody response against the severe acute respiratory syndrome coronavirus 2 (SARS-CoV-2) spike protein, with good production of neutralizing antibodies. Herein, we analyzed differences in the antibody response elicited by inoculation of the Pfizer/BioNTech vaccine through a peptide-based enzyme-linked immunosorbent assay (ELISA) that utilizes synthetic peptides derived from the spike protein in the immuno-adsorbent phase. Immunoreactivity against synthetic peptides was measured at different time points from vaccination and was also correlated with the SARS-CoV-2 neutralizing capacity. Our results indicate that all vaccinated subjects except one show reactive antibodies to at least one peptide at both 30 and 60 days after injection of the first dose. Only one of the 19 analyzed subjects showed no antibody response toward any of the selected peptides, consistently with a lower neutralizing capacity. More importantly, our data showed that the antibody response elicited by inoculation of the two doses of the Pfizer vaccine appears to be qualitatively individual, both in the type of recognized peptides and in the temporal persistence of the antibody response. Together with previous published data, our findings suggest that for effective pandemic control, it is important to constantly monitor the antibody protection in the population, and the assay described here could be a valid tool for this purpose.

## 1. Introduction

More than 18 months after the outbreak of the COVID-19 pandemic due to the SARS-CoV-2 infection, which has severely tested the health and economic structure of the world, there is a shared belief that the introduction of mass vaccination may actually lead to a control of the spread of the virus [1,2]. From this perspective, the design, development, validation, and administration to large populations of different vaccines against COVID-19 in an exceptionally short period of time can be considered one of the greatest achievements of all time in medical history [3]. Among all COVID-19 vaccines, whether prepared or in development, including attenuated, inactivated, subunit, and nucleic acid-based vaccines, those based on mRNA molecules have aroused great interest for their novelty and for the exceptional efficacy observed in the clinical trial phase [2,3].

BNT162b2 developed by Pfizer/BioNTech is a COVID-19 vaccine containing nucleoside-modified messenger RNA encoding the SARS-CoV-2 spike glycoprotein [4]. In healthy adults and young adults older than 16, two 30 μg doses of BNT162b2 elicit high neutralizing titers and robust, antigen-specific T cell responses against SARS-CoV-2, and are 95% effective in preventing severe disease due to virus infection from seven days after the second dose [5,6]. In adolescents of 12–15 years of age, the vaccine’s efficacy was 100% [7].

We previously reported the development of a peptide-based ELISA by selecting seven synthetic peptides from the spike, membrane, and nucleocapsid protein sequences of SARS-CoV-2, which effectively detects the antibody response to SARS-CoV-2 mounted by COVID-19 convalescents [8]. The assay detects a significant difference in the antibody response among single subjects, proving that the antibody responses to SARS-CoV-2 in infected patients appears to have unique repertoire distribution patterns without preference for particular antibody families. Therefore, using the same peptide-based ELISA assay, we verified whether the antibody response elicited by the Pfizer/BioNTech vaccine inoculation was either heterogeneous, similarly to the one elicited by the viral infection, or, alternatively, more homogeneous, being the vaccine based on the expression of the viral spike protein alone.

## 2. Materials and Methods

### 2.1. Study Design and Informed Consent

All study participants (19 individuals: 12 females, 7 males; aged 25–70 years, median 32 years), who also include the authors of this manuscript, are part of the staff of a diagnostic center for COVID-19. Participants received two doses of the Pfizer/BioNTech vaccine (first dose at day 0 and second dose at day 21) and voluntarily underwent venous blood sampling for serological tests at 0, 15, 30, and 60 days after the first dose, in order to monitor the presence of anti-SARS-CoV-2 neutralizing antibodies. All participants signed an informed consent for the anonymized use of the leftover blood sample. None of the participants, except *vax_8*, had a previous history of COVID-19. The study was approved by the Institutional Review Board of Consorzio Sannio Tech (n. 01/2021).

### 2.2. Blood Samples

After clotting, serum was separated using centrifugation for 10 min at 1000 rcf and heat-inactivated for 30 min at 56 °C. Then, 500 μL aliquots were stored at –80 °C. 

### 2.3. ELISA Assay 

The peptides used in this work are reported in Table 1 and ELISA was performed as published elsewhere [8]. Briefly, coating of 96-well high-binding plates (NUNC Maxisorp, Thermo Scientific, USA) with 2 μg/mL of single or pooled peptides in HBSS was performed overnight at 4 °C. Sera samples were diluted in blocking buffer (1:500) and incubated for 1 h at room temperature with continuous agitation. Then, the plates were washed and incubated with 1:60,000 diluted horseradish peroxidase (HRP)-conjugated goat anti-human immunoglobulin G (MERCK KGaA, Darmstadt, Germany). After washing, color-developing reaction was carried out for 15–30 min at room temperature in the dark by adding 70 μL of 1:3 freshly diluted 1-Step^TM^ Ultra TMB-ELISA (Thermo Scientific, Waltham, MA, USA) and stopped with an equal volume of 0.3 M H_2_SO_4_. Absorbance readings at 450 nm were taken using the microplate reader Seac-Sirio-S. Two pre-2019 sera were used as negative controls, while seropositive controls were obtained from two different patients who had been infected with SARS-CoV-2 in the four months prior the study.

Qualitative direct detection of total neutralizing antibodies to SARS-CoV-2 in human serum was performed with a cPass^TM^ SARS-CoV-2 Neutralization Antibody Detection Kit (GenScript Biotech Corporation, Piscataway Township, NJ, USA) according to the manufacturer’s instructions.

## 3. Results and Discussion

Four peptides (Table 1) derived from the spike protein of the SARS-CoV-2 Hu-1 strain (GeneBank: MN908947) were used as the immuno-adsorbent phase in an ELISA. These peptides are generally seen as targets by the antibody response generated following virus infection [8].

**Figure 1:**
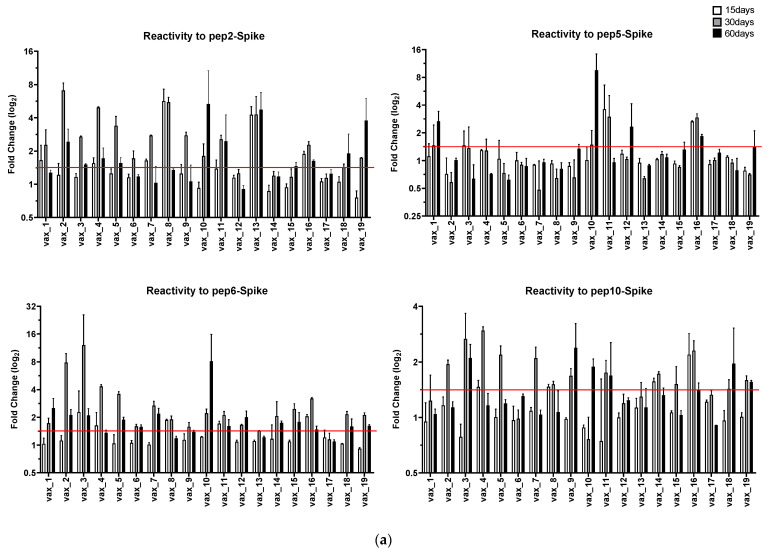

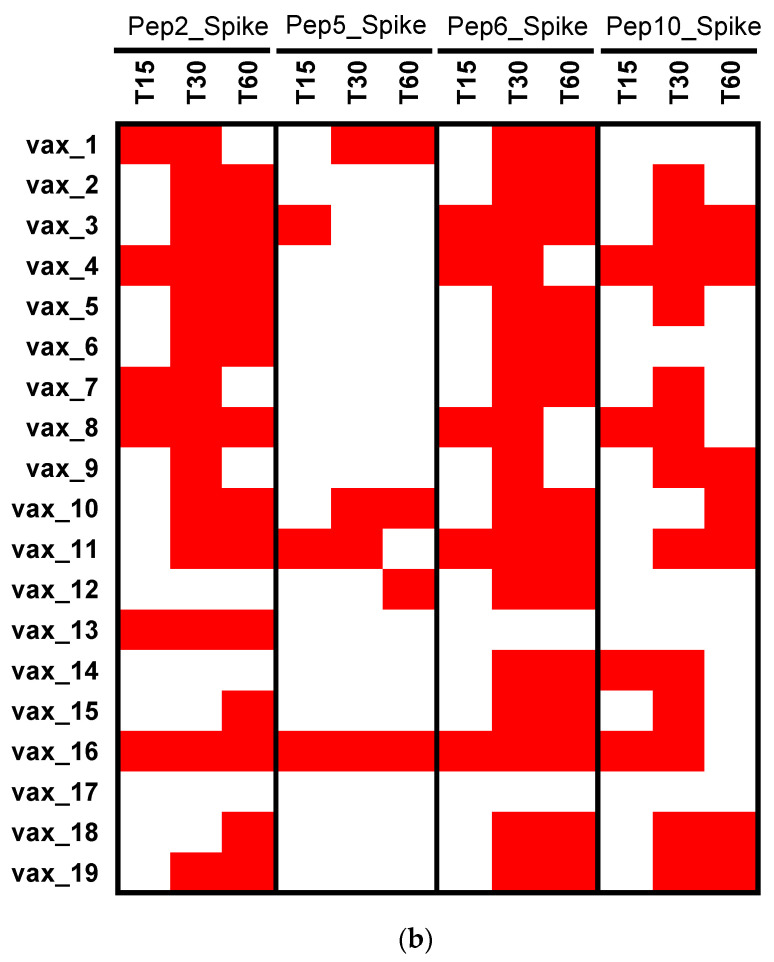
(**a**) IgG immunoreacting with the selected peptides at 15, 30, and 60 days following Pfizer/BioNTech administration of the first dose on day 0 and the second dose on day 21. The antibody response is reported as a log_2_ fold-change compared to day 0. A positive response was arbitrarily scored for a log_2_ fold-change >1.5. (**b**) Summary of IgG sero-reactivities shown in panel (**a**).

Figure 1 shows the IgG antibody response mounted by 19 individuals receiving the first dose of the Pfizer/BioNTech vaccine at day 0 and the second dose at day 21. The humoral response was monitored at days 15, 30, and 60 from day 0. Instead of the signal/cutoff ratio used as a scoring method in previous studies [8], the antibody response is reported as a log_2_ fold-change in the ELISA absorbance readout compared to day 0, which is more useful when analyzing variations through multiple measurements of a biological parameter in the same individual at different time points. In order to better discriminate differences in humoral responses over time, positivity was arbitrarily scored for a log_2_ fold-change >1.5.

**Figure 2:**
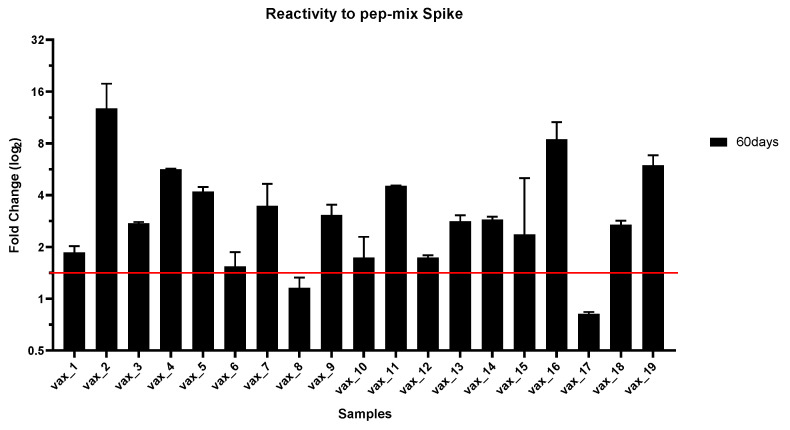
IgG immunoreacting 60 days after administration of the first dose of the Pfizer/BioNTech vaccine using the mixed peptides as the immuno-adsorbent phase. The antibody response is reported as a log_2_ fold-change of ELISA absorbance at day 60 with respect to day 0, when all participants, except *vax_8*, were seronegative to anti-SARS-CoV-2 antibodies.

Reactivity to Pep2_Spike, corresponding to Spike^287–317^, was observed in the sera from all vaccinated subjects, except *vax_12*, *vax_14*, and *vax_17*, in which antibodies against this peptide were under the positivity threshold during the entire study period. However, the antibody response against Pep2_Spike showed different dynamics in different subjects. In fact, many participants resulted negative to the detection of antibodies against this peptide at day 15 after the first dose. In addition, anti-Pep2_Spike antibodies in the vaccinated subjects *vax_1* and *vax_7* are specifically detected as early as 15 days after the first dose, but decreased under the threshold in sera sampled at day 60, i.e., 39 days after the second dose. Positivity to Pep5_Spike, corresponding to Spike^802–819^, was scored only for a few subjects, while many participants never displayed a detectable antibody response to this peptide for the entire period of observation. Pep6_Spike (Spike^601–640^) had the widest reactivity pattern among the vaccinated subjects, with only sera from participants *vax_13* and *vax_17* not reacting to it. A similar diversity of antibody response in the participating subjects was also observable toward Pep10_Spike (Spike^524–598^). Overall, all vaccinated subjects, except participant *vax_17*, showed specific antibodies to at least one peptide at both 30 and 60 days after the injection of the first dose of the Pfizer/BioNTech vaccine. Conversely, subject *vax_17* did not mount an antibody response against any of the peptides used in the assay for the entire period of observation. Indeed, *vax_17* scored negative in an assay performed at day 60 using the mixed peptides as the immuno-adsorbent phase (Figure 2). Specifically, in this last assay, participant *vax_8* also scored below the positivity threshold, due to a past history of COVID-19. In fact, the levels of antibodies were already very high in the serum of *vax_8* collected at day 0; therefore, the vaccination did not result in a large variation on day 60 over day 0 in terms of fold-change.

**Figure 3:**
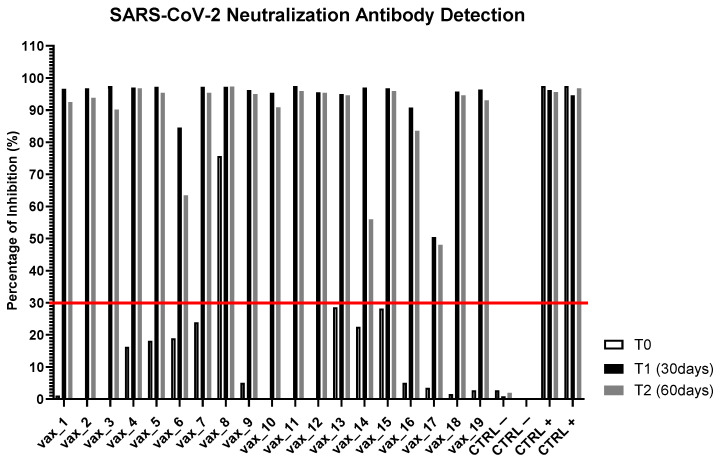
Qualitative direct detection of total neutralizing antibodies to SARS-CoV-2 at day 0 (T0), day 30 (T1), and day 60 (T2) after administration of the first dose of Pfizer/BioNTech.

To verify the reliability of the peptide-based ELISA test, we monitored the presence of neutralizing antibodies to SARS-CoV-2 in the serum of vaccinated subjects using an FDA-approved test. As shown in Figure 3, the sera of all vaccinated subjects at 30 days (T1) and at 60 days (T2) after injection of the first dose (T0) showed an effective neutralizing capacity, with the exception of subject *vax_17*. Interestingly, in two patients, we observed a partial reduction in the percentage of inhibition 60 days after the first dose, suggesting that monitoring SARS-CoV-2 neutralization over time by antibody response could help in defining the level of individual protection, as well as the herd immunity achieved by a population. 

Using an ELISA based on synthetic peptides derived from the spike protein in the immuno-adsorbent phase, we showed that the antibody response elicited by inoculation of the two doses of the Pfizer/BioNTech vaccine appears to be qualitatively individual, both in the type of peptides seen and in the temporal persistence of the antibody response. Overall, all vaccinated subjects except one showed reactive antibodies to at least one peptide at both 30 and 60 days after the injection of the first dose of the Pfizer/BioNTech vaccine. Only one of the 19 analyzed subjects showed no antibody response toward any of the selected peptides, either suggesting a reduced humoral response to the Pfizer/BioNTech vaccine or indicating that further epitopes from the spike proteins could be immunodominant. However, the modest antibody response found in subject *vax_1*7 was confirmed by the poor neutralizing capacity found in its serum at different time points. One caveat to keep in mind is that the antibody positivity threshold was arbitrarily set at a value of log_2_ >1.5 which corresponds to a ~2.8 linear increase in the absorbance value measured by the ELISA. Overall, this short report shows results that are consistent with previously published data, indicating that SARS-CoV-2 mRNA vaccination induces diverse antibodies toward the spike protein [9,10], and that a single dose of mRNA vaccine is enough to elicit rapid humoral responses in seropositive participants [11]. In addition, we observed that in a few subjects, the antibody reactivity to spike’s peptides was reduced at 60 days after inoculation of the first dose, that is, 39 days after injection of the second dose. This means that for effective pandemic control, it is important to constantly monitor the antibody protection in the population, and the assay described here could be a valid tool for this purpose.

An intriguing aspect to further investigate is the T cell-mediated response to synthetic peptides used in this study. Since the beginning of the pandemic, seroconversion in convalescent patients has been extensively monitored due to the technical ease of specific antibody detection. Nonetheless, although the analysis of T cell immunity may be more challenging to carry out on a population-wide scale, it is crucial for patients who do not mount an efficacious antibody response toward virus infection and/or vaccination [12,13]. More specifically, it would be interesting to evaluate whether the synthetic peptides described here and in our previous work could recapitulate, or partially represent, the portfolio of viral epitopes presented to T cells after both infection and vaccination, and if any reactivity might be informative of a long-term effective immune protection. In addition, they could also be useful for evaluating potential T cell cross-reactivity in unexposed individuals due to endemic diffusion of cold coronaviruses [12].

From such a perspective, the development of synthetic peptide-based clinical tests aimed to measure T cell immunity may also guide physicians in determining, on the one hand, when booster vaccination is appropriate and, on the other hand, whether vaccination strategies are able to contain the spread of variant forms of SARS-CoV-2 [13,14]. Overall, collecting data on the ability of both antibodies and T lymphocytes to recognize SARS-CoV-2 peptides from convalescent and/or vaccinated individuals would allow to obtain more consistent and complete information on long-term protection after the ongoing vaccination campaigns and toward newly recognized variants of concern [14]. 

## Figures and Tables

**Table 1:** List of peptides used for this study.

Peptide	Sequence	Position
Pep2_Spike	DAVDCALDPLSETKCTLKSFTVEKGIYQTSN	287–317
Pep5_Spike	FSQILPDPSKPSKRSFIE	802–819
Pep6_Spike	GTNTSNQVAVLYQDVNCTEVPVAIHADQLTPTWRVYSTGS	601–640
Pep10_Spike	VCGPKKSTNLVKNKCVNFNFNGLTGTGVLTESNKKFLPFQQFGRDIADTTDAVRDPQTLEILDITPCSFGGVSVI	524–598

## Data Availability

Data are available on request from the corresponding authors.

## Abbreviations

COVID-19	Coronavirus disease 2019
mRNA	Messenger ribonucleic acid
SARS-CoV-2	Severe acute respiratory syndrome coronavirus 2
ELISA	Enzyme-linked immunosorbent assay
